# The Oncologic Outcomes of Bilateral Central Lymph Node Dissection in Unilobar Papillary Thyroid Cancer and Its Risks: A Prospective Cohort Study

**DOI:** 10.7759/cureus.65443

**Published:** 2024-07-26

**Authors:** Mohamed Y Abuahmed, Rahel Rashid, Waleed A Aboelwafa, Yasser M Hamza

**Affiliations:** 1 UGI Surgery, Wirral University Teaching Hospital, Liverpool, GBR; 2 General and Colorectal Surgery, Arrowe Park Hospital, Wirral, GBR; 3 Head and Neck Surgery, Alexandria University Teaching Hospital, Alexandria, EGY; 4 Head and Neck Surgery, Alexandria University Teaching Hospitals, Alexandria, EGY

**Keywords:** hypocalcemia, lymph node metastasis, central neck dissection, papillary thyroid cancer, thyroid gland

## Abstract

Background

Indications for performing a prophylactic central neck dissection (pCND) in papillary thyroid cancer (PTC) remain controversial. Thyroidectomy and central neck dissection (CND) are often recommended in all cases with proven differentiated thyroid cancer (DTC) and clinically positive lymph nodes (LNs), as well as in high risk for micro-metastasis patients with T3-T4 tumors or established metastatic nodes in the lateral compartments.

Aims

The aims of this study were to ascertain the role of performing bilateral central LN dissection in unilobar PTC in improving the oncological outcomes and outline the risks involved.

Methods

This was a department-based, prospective cohort study. We included all 20 patients who had unilobar PTC and underwent total thyroidectomy with bilateral CND. A postoperative histopathological analysis was used to identify metastatic central LNs.

Results

Twenty total thyroidectomies plus bilateral CNDs were performed, of which 10 were prophylactic bilaterally (those with N0), and all 20 were prophylactic on the contralateral side of PTC. Conventional risk factors (age, tumor size, and extrathyroidal extension) were not associated with performing a pCND. The presence of unilobar PTC by preoperative FNAC was the only factor associated with performing bilateral CND. Positive ipsilateral LNs were retrieved in 55% of CNDs, while positive contralateral LNs were retrieved in only 15% of the patients.

Conclusions

The incidence of contralateral cervical LN metastasis in patients with unilateral PTC is low, while there is clear evidence of postoperative morbidity from routine contralateral CND in unilobar PTC. Contralateral CND in patients with unilobar PTC may be reserved for high-risk patients: males, those aged ≤45 years, tumors larger than 1.0 cm, and cases with extrathyroidal extension and micro-calcification on ultrasound.

## Introduction

Thyroid cancer is a common endocrine malignancy, with the peak incidence typically observed between 40 and 50 years of age, especially in women, who are three times more likely to be affected than men [1,2]. Despite its increasing incidence over the past decades, thyroid cancer generally carries a favorable prognosis, with an overall five-year survival rate exceeding 98% [3]. The effectiveness of available treatments, including surgical interventions, radioactive iodine (RAI) therapy, and long-term thyroid-stimulating hormone suppression, contributes to the high survival rates observed in patients with thyroid cancer [4]. These advancements in diagnosis and treatment have significantly improved outcomes for individuals diagnosed with thyroid cancer, highlighting the highly treatable nature of this disease and the positive prognosis associated with it.

While most papillary thyroid micro-carcinoma (PTMCs) are indolent and have a favorable prognosis, some may exhibit invasive characteristics, with central lymph node (LN) metastasis being considered a risk factor for invasiveness [5]. Despite the challenges in distinguishing between indolent and invasive PTMCs, the overall prognosis for PTMCs remains good. The changing trends in the management of thyroid nodules may have influenced the diagnosis of small, indolent tumors, impacting the observed incidence rates.

The latest American Thyroid Association (ATA) guidelines recommend total thyroidectomy for tumors larger than 4 cm or with high-risk features like family history, prior neck irradiation, extra thyroid extension, multifocality, and LN involvement, necessitating extensive surgical interventions [6]. LN metastasis is common in papillary thyroid cancer (PTC), affecting 20-50% of individuals, especially in the central neck compartment (level VI), posing a significant risk for local recurrence [7]. When LN metastases are confirmed, therapeutic dissection of both central (levels VI and VII) and lateral neck compartments (levels II-V) is typically advised to manage the spread effectively [8]. This comprehensive approach aims to reduce the risk of recurrence and improve patient outcomes by addressing both central and lateral LN involvement in PTC cases.

Prophylactic central neck dissection (pCND) in PTC patients without clinically suspicious LN metastases remains a topic of debate. Studies have shown conflicting results regarding the impact of pCND on locoregional recurrence and long-term outcomes. While some research suggests that pCND does not significantly reduce locoregional recurrence rates [9], others indicate that omitting pCND can be an independent risk factor for recurrence in the central compartment [10]. Additionally, the presence of metastases in central LNs is associated with primary tumor size, emphasizing the importance of individualized treatment strategies [11]. The use of near-infrared fluorescence imaging has shown promise in reducing the risk of complications associated with central neck dissection (CND), potentially influencing future recommendations regarding pCND [12].

The 2009 ATA guidelines recommended pCND for advanced primary tumors (T3 or T4) and clinically involved lateral neck nodes (cN1b) or when needed for further therapy planning [10,11]. Post-guideline publication, the surgical community debated the value of pCND in high-risk PTC patients. Metastatic central compartment LNs are linked to gender, primary tumor size, BRAF mutations, extrathyroidal extension, and lateral cervical LN metastases, prompting some authors to consider these factors as indications for pCND [12-14]. The debate revolves around the necessity of pCND in high-risk PTC cases and the association of metastatic central compartment LNs with various risk factors.

Studies have shown conflicting results regarding the impact of pCND on overall survival in well-differentiated thyroid cancer (WDTC) [12,15]. While some small studies suggest that cervical LN metastases may affect survival [15], larger epidemiologic studies do not consistently support this notion [16]. A recent randomized controlled trial focusing on pCND in PTC found that patients undergoing pCND required fewer repeat doses of RAI but had significantly higher rates of permanent hypoparathyroidism [17]. Due to these conflicting findings, many surgeons have developed individualized algorithms for determining when to perform pCND and the extent of dissection (unilateral vs. bilateral CND) [18].

Therefore, the primary objective of this study was to identify the oncologic benefits of bilateral CND in unilateral PTC, i.e., to assess the effectiveness of pCND on the contralateral side. The secondary objective was to assess the risks associated with this procedure.

This article was previously presented as an oral presentation at the 2024 European Association of Endoscopic Surgery (EAES) Annual Congress in Maastricht, the Netherlands, on June 13, 2024.

## Materials and methods

This study employed a prospective, observational design to investigate the preoperative assessment, operative procedures, and postoperative management in patients with unilobar PTC undergoing total thyroidectomy with bilateral CND.

All patients who had unilobar PTC and were scheduled for total thyroidectomy with bilateral CND at Alexandria University Teaching Hospital, Alexandria, Egypt, between July 2022 and June 2023 were considered for inclusion in this study. Excluded were the patients with bilateral PTC, benign thyroid disease, and metastatic thyroid cancer.

The primary outcome was oncological clearance detected by postoperative histopathology. The secondary outcome is postoperative complications associated with the procedure.

**Figure 1:**
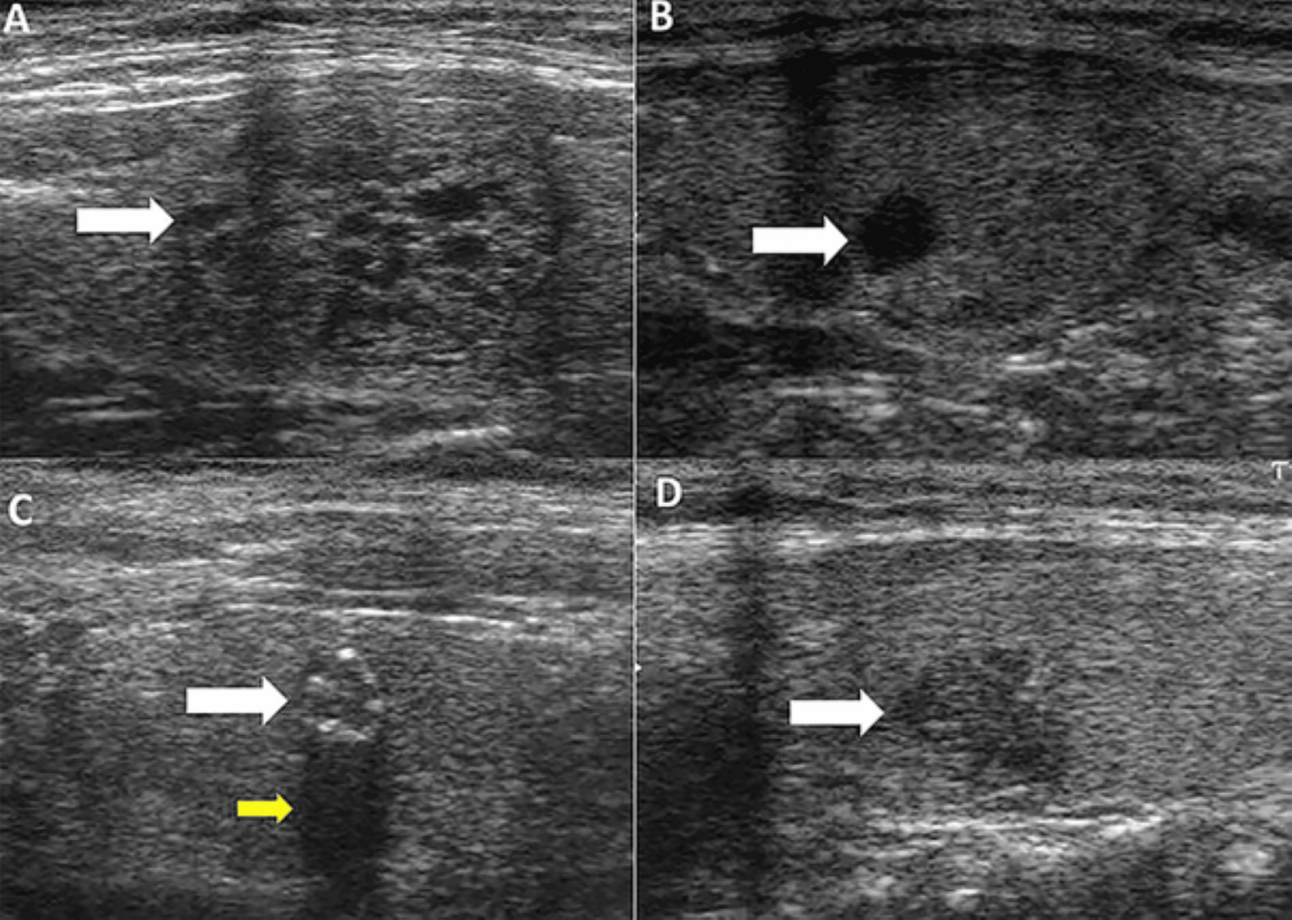
B-mode ultrasound characteristics of thyroid nodules (A) Isoechoic well-defined sponge-like appearance nodule (white arrow). (B) Anechoic well-defined cystic nodule (white arrow). (C) Hypoechoic solid well-defined nodule with micro-calcification (white arrow), causing acoustic shadowing (yellow arrow). (D) Hypoechoic solid nodule with irregular margin (white arrow).

All patients underwent comprehensive preoperative assessment, including complete history-taking, thorough clinical examination, and assessment of vocal cord function by indirect laryngoscopy. Patients with central neck swelling (goiter) with or without lateral neck swellings were included. All patients underwent a series of laboratory investigations, including complete blood picture, serum urea and creatinine, liver function tests (LFTs), electrocardiogram (ECG), and echocardiogram (if aged over 50 years). Coagulation profile (prothrombin time, partial thromboplastin time, and INR), thyroid function tests (free triiodothyronine [FT3], free thyroxine [FT4], and thyroid-stimulating hormone [TSH]), and basal thyroglobulin levels were also assessed. Patients with normal laboratory investigations and thyroid function tests were included. All patients underwent an ultrasound scan (USS) of the neck preoperatively. Patients with proven unilobar suspicious thyroid nodules with or without ipsilateral suspicious LNs were included (Figure 1) [19].

All patients unilaterally had FNAC from suspicious thyroid nodule(s). Only patients with Bethesda IV, V, and VI were included in this study [20]. This diagnostic technique is effective, minimally invasive, and economical, with a very low chance of seeding tumor cells along the needle track. This method may reach the diagnosis with a sensitivity of 82-96% and a specificity of 92-100% for metastatic lymphadenopathy if applied by a skilled interventional radiologist. Table 1 summarizes the Bethesda system that was followed.

**Table 1:** The Bethesda System for Reporting Thyroid Cytopathology with implied risk of malignancy and recommended clinical management AUS, atypia of undetermined significance; FLUS, follicular lesion of undetermined significance; FNA, fine needle aspiration

Diagnostic category	Number of nodules	Relative frequency (%)
(I) Nondiagnostic	69	10.1
(Il) Benign	469	68.8
(III) AUS/FLUS	85	12.4
(IV) Suspicious for follicular neoplasm	20	2.9
(V) Suspicious for malignancy	18	2.6
(VI) Malignant	28	4.1

All patients underwent total thyroidectomy under general anesthesia with endotracheal intubation. CND was performed through a standardized neck collar incision. Skin flaps were raised upward to the level of the thyroid notch and downward to the sternal notch. The midline between the strap muscles was opened through a vertical incision from the thyroid notch to the sternal notch. Parathyroid glands (PTGs) were marked with a small vascular metal clip or sutured away from their blood supply to aid identification during CND. Prelaryngeal and pretracheal dissections were performed as described in the literature. The wound was closed with subcuticular sutures with suction.

Postoperative monitoring was done for all patients, including assessment of hypocalcemia clinically and by laboratory investigations. Surgical site, voice, airway patency, and swallowing ability were also assessed postoperatively.

Specimens acquired during surgery, including the thyroid gland and central LNs, underwent histopathological examination. LNs were divided into ipsilateral and contralateral groups based on their location relative to the trachea. Pathological analysis included assessment of LN involvement, presence of metastasis, and extranodal infiltration.

The short-term postoperative assessment included monitoring of total and ionized calcium levels. Long-term follow-ups will be done with thyroglobulin levels.

Data analysis was done using the Jamovi statistical analysis software program to calculate the frequency and percentage of patients who had PTC and underwent total thyroidectomy with bilateral CND. Moreover, the frequency and percentage of patients who had postoperative complications were also analyzed using the same statistical analysis program.

## Results

The study was conducted between July 2022 and June 2023. A cohort of 20 patients was analyzed. Notably, the majority were females (16 patients), comprising 80% of the sample, with over half of the patients being 40 years old or older, and the median age was 41 (IQR, 26.5-49) (Table 2).

**Table 2:** Distribution of the studied cases according to demographic data

Demographic data	Number	%
Gender		
Male	4	20
Female	16	80
Age (years)		
<40	9	45
≥40	11	55

Among the reported symptoms, midline neck swelling was the most common complaint in 19 patients, representing 95% of the cases, followed by compressive manifestations such as dyspnoea and dysphagia in 12 patients (60%), and finally, lateral neck swelling happened in 10 patients (50%) (Table 3).

**Table 3:** Distribution of the studied cases according to the complaint

Complaint	Number	%
Complaint midline neck swelling	19	95.0
Lateral neck swelling	10	50.0
Compression manifestations	12	60.0

According to local examination, 19 of 20 (95%) patients had palpable goiter, while only 11 of them (55%) had a palpable LN (Table 4).

**Table 4:** Distribution of the studied cases according to local examination of lymph nodes

Local examination of lymph node level	Number	%
Palpable goiter	19	95
Palpable lymph node		
Right	5	25
Left	6	30
Lymph node level		
II	4	20
III	8	40
IV	6	30
V	2	10

**Figure 2:**
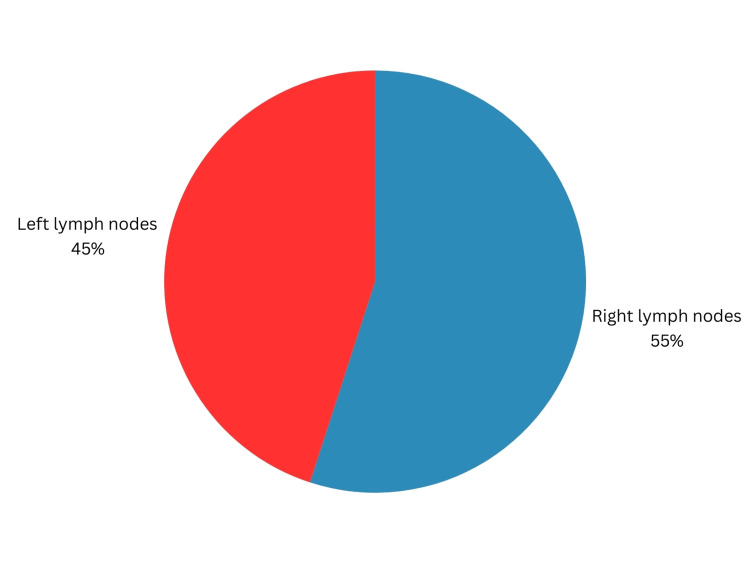
Distribution of the studied cases according to the side of lymph node involvement

Of the patients with palpable LNs, five patients (45%) had palpable LN on the right side, while six patients (55%) had palpable LN on the left side (Figure 2).

Regarding preoperative USS, 10 patients (50%) had multinodular goiter (MNG), while only two patients (10%) had diffuse goiter, three patients (15%) had right solitary thyroid nodule (STN), and five patients (25%) had left STN (Table 5).

**Table 5:** Distribution of the studied cases according to ultrasound scan of the thyroid gland STN, solitary thyroid nodule; MNG, multinodular goiter

Ultrasound result of STN	Number	%
Diffuse goiter	2	10
MNG	10	50
STN		
Right	3	15
Left	5	25

More importantly, regarding ultrasound-detected LN metastasis in the neck, 13 patients (65%) were N1, while seven patients (35%) were N0. Of the N1 patients, two patients (10%) had detected metastatic central LN by ultrasound (Table 6).

**Table 6:** Distribution of the studied cases according to ultrasound examination of cervical lymph nodes

Lymph nodes	Number	%
Right	5	25
Left	7	35
Stage		
N0	7	35
N1	13	65
Level		
II	5	25
III	8	40
IV	7	35
V	3	15
VI	2	10

Preoperatively, FNAC was done for all patients. Twelve patients (60%) had malignant goiter, eight patients (40%) had Bethesda V, and four patients (20%) had Bethesda VI.

Additionally, four patients (20%) had metastatic level II, five patients (25%) had metastatic level III, two patients (10%) had metastatic level IV, and none (0%) had metastatic level V (Table 7).

**Table 7:** Distribution of the studied cases according to preoperative FNAC of thyroid and lymph nodes FNAC, fine needle aspiration cytology

Preoperative FNAC thyroid gland	Number	%
Thyroid gland		
Bethesda I	1	5
Bethesda IV	7	35
Bethesda V	8	40
Bethesda VI	4	20
Lymph nodes		
II		
Negative	16	80
Metastatic	4	20
III		
Negative	15	75
Metastatic	5	25
IV		
Negative	18	90
Metastatic	2	10
V		
Metastatic	0	0

Preoperative assessment of vocal cords revealed that 19 of 20 patients (95%) had bilaterally freely mobile vocal cords.

Postoperative complications were recorded clinically for all patients after the operation. The most common complication was voice changes, where seven (35%) patients suffered from temporary voice changes and one (5%) patient suffered from permanent voice changes.

**Figure 3:**
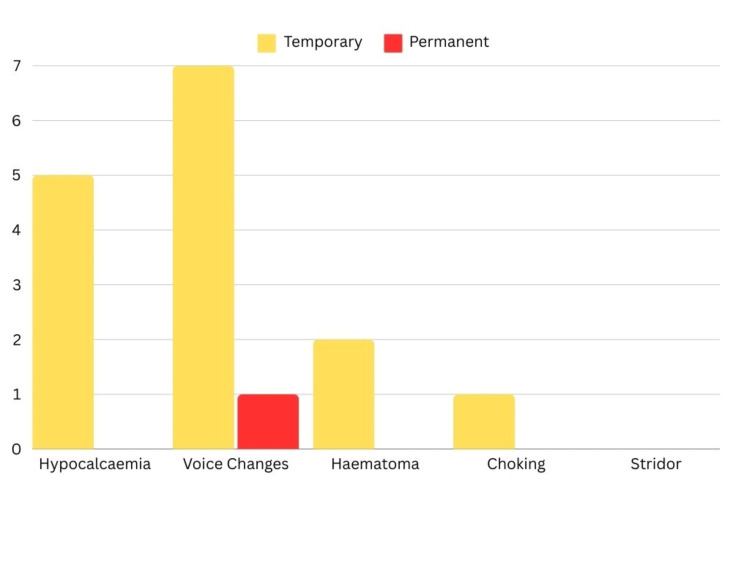
Distribution according to postoperative complications

The second most common complication was hypocalcemia, where five patients (25%) suffered from temporary manifestations like circumoral, hand and feet tingling with positive Chvostek sign. Other complications were hematoma (two patients) and choking (one patient). No patient suffered from stridor. Figure 3 outlines the incidence of postoperative complications.

**Figure 4:**
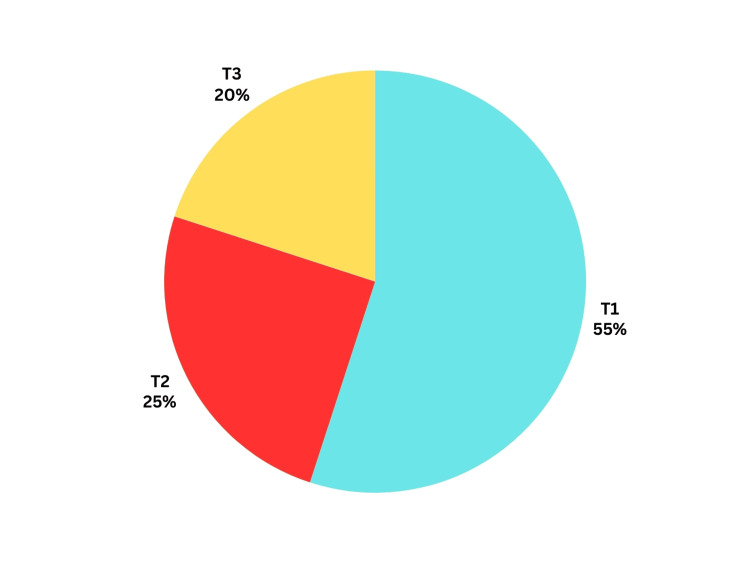
Distribution of studied cases according to postoperative T-staging

Postoperative histopathological staging showed that 11 patients were T1 stage (55%), five patients were T2 stage (25%), and only four patients were T3 stage (20%) (Figure 4).

More importantly, the postoperative histopathology of central LNs revealed that 11 patients (55%) had only ipsilateral central LN involvement, three patients (15%) had both ipsilateral and contralateral LN involvement, and six patients (30%) had neither side involved (Table 8).

**Table 8:** Distribution of studied cases according to CND involvement CND, central neck dissection

	Level	Count	Total	Proportion
CND involvement	Ipsilateral	11	20	55%
	Not involved	6	20	30%
	Contralateral	3	20	15%

## Discussion

This study underscores the clinical presentation, diagnostic findings, and postoperative outcomes of patients undergoing thyroidectomy and bilateral CND, providing valuable insights into the management and implications of PTC.

In the evaluation of contralateral central LN dissection in patients with unilateral/unilobar PTC, several key findings emerge from the literature. Studies have shown that the prevalence of occult contralateral neck metastases in lateralized oral squamous cell carcinoma with ipsilateral LN metastasis is low, suggesting that routine contralateral elective neck dissection may not be necessary for this cohort [21]. Additionally, the effectiveness of pCND in clinically node-negative PTC patients indicates a lower local recurrence rate with the procedure, albeit with higher risks of permanent hypocalcemia and transient hypoparathyroidism, emphasizing the importance of weighing oncologic benefits against potential complications [22]. Furthermore, machine learning algorithms have been developed to predict the risk of central LN metastasis in cN0 PTC patients, aiding in individualized treatment decisions and enhancing surgical outcomes [23]. The research findings from various studies on PTC suggest that performing bilateral central LN dissection for radiologically or pathologically confirmed unilateral PTC may not provide significant oncologic benefits [9,24]. While the proportion of patients with positive metastasis in contralateral central LNs was statistically insignificant at 15%, indicating limited benefit from bilateral dissection [25], there was a notable increase in postoperative complications, including voice changes and hypocalcemia manifestations affecting up to 35% of patients, with some experiencing temporary voice alterations [9,26]. These findings highlight the importance of carefully weighing the risks and benefits of extensive LN dissection in PTC patients to optimize treatment outcomes and minimize complications. The research on PTC patients emphasizes the critical evaluation of risks and benefits related to contralateral central LN dissection [9,27-29]. Studies have shown that prophylactic CND in PTC patients with clinical LN-negative (cN0) status can be beneficial, especially for high-risk individuals [30]. Factors such as tumor size, multifocality, and extrathyroidal extension play significant roles in determining the need for CND. Additionally, the presence of contralateral paratracheal LN metastasis has been linked to contralateral central LN metastasis in unilateral PTC cases, guiding the extent of LN dissection. These findings align with existing literature, supporting the individualized and precise approach to treatment decisions in PTC patients to optimize outcomes while minimizing risks [30].

Despite the valuable insights gained from this study, limitations such as small sample size and selective participant criteria must be acknowledged. Moving forward, larger and more diverse studies are warranted to enhance the generalizability of our findings.

## Conclusions

In conclusion, while contralateral central LN dissection may not confer significant oncologic benefits in unilateral PTC, it may be warranted in high-risk patients, necessitating careful patient selection to minimize postoperative morbidity. Complications can be severe enough to affect the patient’s quality of life and sometimes cause patient’s mortality, although this did not occur during our study. Further RCTs are required to balance the oncological benefits with the postoperative complications.
